# Peptide and Peptide-Dependent Motions in MHC Proteins: Immunological Implications and Biophysical Underpinnings

**DOI:** 10.3389/fimmu.2017.00935

**Published:** 2017-08-07

**Authors:** Cory M. Ayres, Steven A. Corcelli, Brian M. Baker

**Affiliations:** ^1^Department of Chemistry and Biochemistry, University of Notre Dame, Notre Dame, IN, United States; ^2^Harper Cancer Research Institute, University of Notre Dame, South Bend, IN, United States

**Keywords:** MHC, peptide, flexibility, dynamics, antigenicity

## Abstract

Structural biology of peptides presented by class I and class II MHC proteins has transformed immunology, impacting our understanding of fundamental immune mechanisms and allowing researchers to rationalize immunogenicity and design novel vaccines. However, proteins are not static structures as often inferred from crystallographic structures. Their components move and breathe individually and collectively over a range of timescales. Peptides bound within MHC peptide-binding grooves are no exception and their motions have been shown to impact recognition by T cell and other receptors in ways that influence function. Furthermore, peptides tune the motions of MHC proteins themselves, which impacts recognition of peptide/MHC complexes by other proteins. Here, we review the motional properties of peptides in MHC binding grooves and discuss how peptide properties can influence MHC motions. We briefly review theoretical concepts about protein motion and highlight key data that illustrate immunological consequences. We focus primarily on class I systems due to greater availability of data, but segue into class II systems as the concepts and consequences overlap. We suggest that characterization of the dynamic “energy landscapes” of peptide/MHC complexes and the resulting functional consequences is one of the next frontiers in structural immunology.

## Introduction

Presentation of peptides by class I MHC (MHC-I) proteins to T cell receptors (TCRs) is a key component of cellular immunity. The first structures of peptide/MHC-I structures illustrated how peptides were presented by MHC-I proteins, lying in an extended form embedded within a binding groove formed by two flanking α helices and a β sheet floor (1). The general architecture of peptide/MHC-I complexes is widely recognized, with representations found within thousands of reviews, research publications, and textbooks ranging from general biology to advanced immunology. The solution of peptide/MHC-I structures answered fundamental questions in immunology, including how a single receptor can simultaneously recognize both self (MHC) and non-self (peptide) in antigen recognition (2). The subsequent solution of structures of complexes between TCRs and peptide/MHC-I complexes illustrated at an atomic level how simultaneous recognition of self/non-self occurs (3, 4).

**Figure 1:**
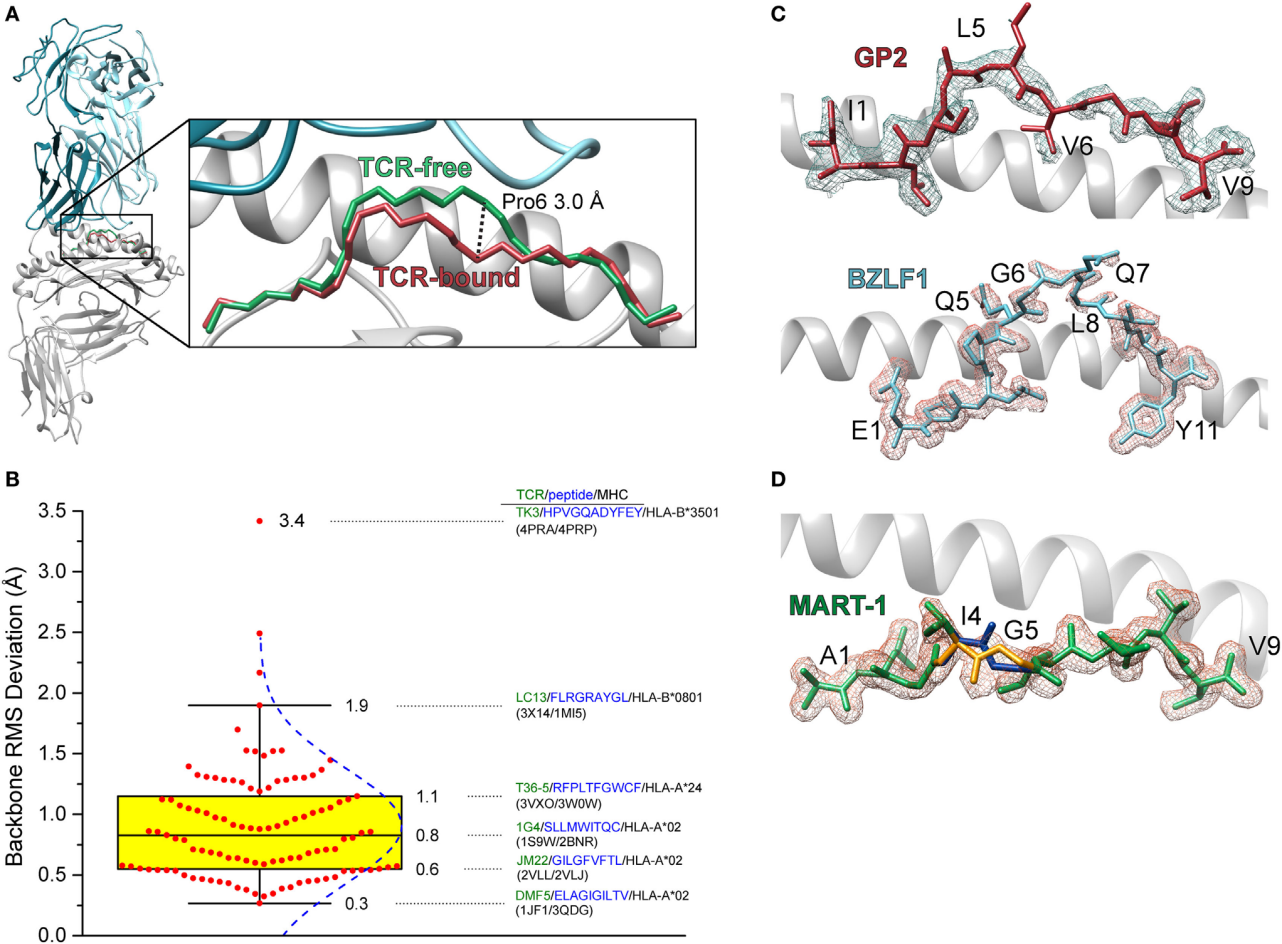
Conformational changes in peptides and heterogeneity in peptides bound to class I MHC (MHC-I). **(A)** Illustration of the change in the backbone of the Tax_11–19_ peptide bound to HLA-A2 upon binding of the A6 T cell receptors (TCR). The conformational change is centered upon amino acids 6 and 7, with a maximal displacement of 3 Å occurring at the α carbon of position 6. The root mean square (RMS) deviation for contiguous peptide backbone atoms between TCR-free and TCR-bound is 1.3 Å. **(B)** Statistics of peptide conformational changes that occur upon TCR binding for all peptide/MHC-I complexes for which TCR-free/bound structures exist in the Protein Data Bank. The figure shows a Box plot of peptide backbone RMS deviations between free and bound structures. Individual values are indicated by red dots, and the interquartile range between the 75th and 25th percentiles indicated in yellow. Whiskers extend to the furthest values that lie within the 75th and 25th percentile value ±1.5× the interquartile range. RMS deviations are binned according to the Freedman–Diaconis rule for a total of 12 bins (12). The blue dashed curve indicates the population distribution. Complexes and TCR-free/bound PDB codes are provided for the 25th, 50th, and 75th percentile values, as well as for the points that demarcate the whiskers and for the system which displayed the largest peptide conformational change upon binding. **(C)** Weak electron density for the triply modified 10-mer peptide GP2 bound to HLA-A2 (top) and the 11-mer peptide BZLF1 bound to HLA-B35 (bottom) (13, 14). Gaps in the density show regions where conformational heterogeneity is likely to exist. Density is calculated from a 2*F_o_*−*F_c_* map and contoured at 1σ. **(D)** Multiple conformations of the anchor modified MART-1_27–35_ ALG nonameric peptide bound to HLA-A2 (15). The electron density was sufficiently clear to allow refinement of the backbone at positions 4 and 5 in two different conformations (blue and gold regions).

The first high-resolution crystal structure of a TCR-peptide/MHC-I complex was that of the A6 TCR bound to the HTLV-1 Tax_11–19_ antigen presented by HLA-A2 (3). Comparison of the TCR-bound complex with that of the free peptide/MHC-I revealed that the peptide undergoes a conformational change upon TCR binding, centered around the central core—the peptide is essentially “squished” into the binding groove by the receptor (5) (Figure 1A). Many subsequent structures demonstrated that peptide conformational changes frequently occur upon TCR binding (Figure 1B). The regularity of this occurrence is likely related to the fact that in MHC-I complexes peptides are usually not flat within the binding groove, but bulge due to the tethering of the N- and C-terminal residues, with the degree of bulging increasing with peptide length (6–9). Peptides can also be “pulled” away from the binding groove in response to TCR binding, an occurrence which could be related to the identity of the primary anchor residues (10, 11).

In some crystal structures of peptide/MHC-I complexes, peptides are poorly refined in the binding groove, with side chains and even backbones lacking electron density (Figure 1C). There can be multiple reasons for weak or missing electron density in protein structures, such as poor crystal morphology or even the existence of multiple peptides in one crystal, as was the case in the very first structure of HLA-A2 (16). Another reason for poor electron density is structural heterogeneity, stemming from the existence of multiple peptide conformations or the interconversion between different conformations on the timescale of the X-ray diffraction experiment. Such heterogeneity was demonstrated in an early experiment with the GP2 HER-2/neu epitope, which had missing density in the peptide center when bound to HLA-A2 (17). In other cases, electron density is clear enough to identify peptides in multiple conformations (15) (Figure 1D).

What do binding-induced conformational changes and structural heterogeneity have in common? They are both indicative of molecular motion, or protein dynamics. Proteins are not static molecules, and their atoms move independently and collectively over a wide range of timescales. Peptide/MHC complexes are no exception, and peptide conformational changes, weak electron density, and structural heterogeneity give some insight into the influence that motion can have in antigen recognition. Below, we outline how peptide motion can be important in influencing antigenicity and suggest how it may be considered in efforts to predict and even manipulate immune recognition.

## Theoretical Considerations of Protein Motion

Although not long considered in structural and molecular immunology, the study of protein dynamics has a long and rich history. The first computational studies of protein motion were performed in the 1970s [e.g., Ref. (18)] and experimental studies using various forms of spectroscopy [fluorescence, nuclear magnetic resonance (NMR)] date to the 1960s (19). The question of how protein motion drives biological function was considered as early as 1972 in discussions about conformational changes occurring in hemoglobin as it performs its physiological functions (20).

**Figure 2:**
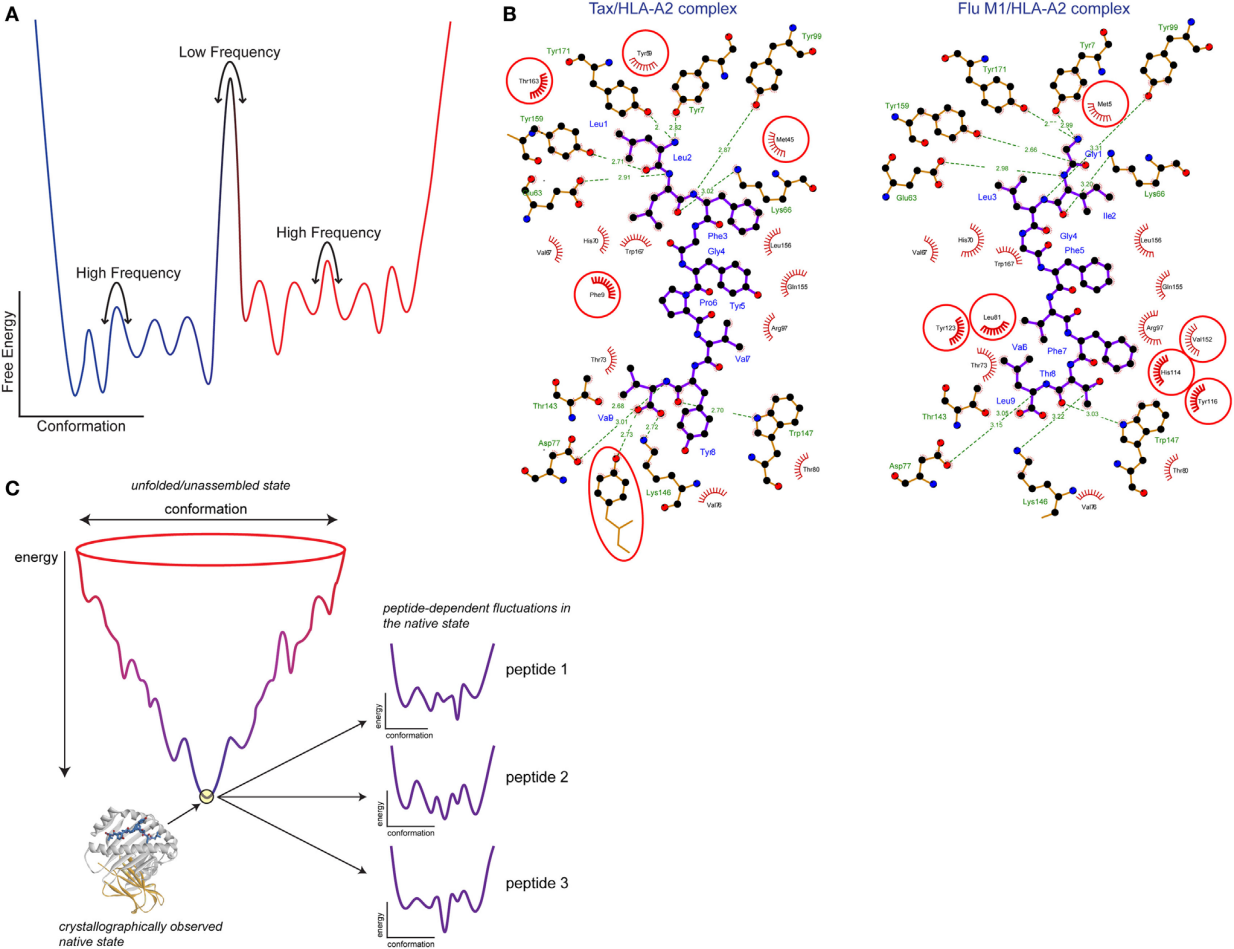
Free energy landscapes and peptide tuning of the class I MHC (MHC-I) energy landscape. **(A)** Schematic showing the variation in protein-free energy with conformation. Atomic coordinates are along the *x* axis and free energy is along the *y* axis. Wells along the conformation axis represent different structural substates separated by barriers. The heights of the barriers yield the rates at which the protein moves between (or samples) different conformations. Low barriers translate into rapid, high-frequency motions, whereas high barriers translate into slower, low-frequency motions. The number and energy levels of the structural substates separated by the barriers gives the protein entropy. **(B)** Peptide tuning of the MHC-I energy landscape. Ligplot analysis (25) of the interactions the Tax and Flu M1 peptides make with HLA-A2. Peptides are shown with purple bonds and contacting MHC residues are indicated. Interactions formed by hydrophobic atoms are shown with red hashes. Hydrogen bonds are shown with green lines with distances indicated. HLA-A2 residues making significant interactions in one complex but not the other are circled in red. **(C)** Illustration of how peptides can tune the MHC-I energy landscape. The image shows a traditional folding funnel, with the native peptide/MHC-I architecture at the bottom of the funnel. Zooming into the tip of the funnel reveals the energy landscape of the assembled complex, as diagrammed in panel **(A)**. Due to the different interactions formed by different peptides as shown in panel **(B)**, the energy landscape is altered, changing MHC-I protein dynamics. Figure adapted from Ref. (26) and used by permission.

Often, protein dynamics that influence molecular recognition are described using terms such as induced fit, preexisting equilibrium, or conformational selection. Induced fit is commonly used to describe structural differences that are apparent between free and bound structures, whereas preexisting equilibrium or conformational selection is often used to describe rapid motion that occurs in an un-bound protein. Although used to describe distinct scenarios, each of these terms ultimately reflects the influence of motion (21, 22). The distinctions boil down to timescales, or the rates at which proteins exhibit flexibility and undergo conformational changes. Timescales and rates in return relate to the heights of free energy barriers between conformations and the frequencies with which these barriers are overcome. In that sense, classical induced fit and preexisting equilibria/conformational selection mechanisms reflect extremes on a continuum—slow, or low frequency motion with high barriers for the former, and rapid, high frequency motion with low barriers for the latter (Figure 2A). The barriers between different structural states can be modulated by changes in the molecular environment, contributing to “induced fit” changes occurring during binding—but nonetheless, induced fit inherently reflects a propensity to move. In some cases, assignment of structural changes to either induced fit or conformational selection can be clear, such as seen with recognition of peptides by the A6 and G10 TCRs (23, 24). More often though, when induced fit, structural heterogeneity or poor electron density is indicated from crystal structures, the heights of the barriers can only be guessed at, so where on the continuum the associated protein motions lie is often unknown.

How can protein motions—fast, slow, or intermediate—influence molecular recognition, such as occurs between a TCR and peptide/MHC complex? To form the most stable complexes (i.e., those with the lowest free energy), proteins need to optimize shape and chemical complementarity within the protein–protein interface. If motions are required to achieve this, then these motions can limit the rate at which complexes form, in turn limiting the overall affinity of the complex. An example is found in the antibody maturation process, during which mutations are introduced that remove conformational heterogeneity and its associated motion from the antibody binding site (27). Accordingly, one signature of antibody maturation is an increase in the rate of association rates as maturation proceeds, strengthening binding (28).

A related mechanism is associated with the population of multiple conformational states—the various “wells” between the barriers in Figure 2A. Conformational heterogeneity is directly related to entropy: the more states that are populated, the greater the entropy. If heterogeneity is reduced upon binding as is usually expected, then structural heterogeneity will increase the entropic cost for binding, and affinity will weaken. Again using antibodies as an example, affinity maturation is associated with a reduction in antibody conformational heterogeneity and lower entropic costs for binding (27).

Consider TCR recognition of a peptide/MHC-I complex in the context of the above discussion. A peptide that must move to optimize fit with a TCR, such as the Tax_11–19_ antigen presented by HLA-A2 in Figure 1A, will result in slower binding compared to a more rigid peptide presented in an optimal conformation. Indeed, recognition of the Tax_11–19_/HLA-A2 complex by the A6 and B7 TCRs proceeds with association rates in the range of ~0.5 × 10^6^ M^−1^ s^−1^, orders of magnitude slower than the diffusion controlled limit (29). Other TCR binding reactions associated with peptide conformational changes occur with similar rates, or even slower (although it is important to note that TCR motional properties can also influence association rates, confounding the assignment of slow association rates purely to peptide conformational changes) (30). Clear demonstration of entropic penalties associated with peptide conformational changes or motion are more difficult to find given the varied contributions to binding entropy changes, but many TCRs bind with unfavorable entropy changes (31).

## Assessing Peptide Motions in MHC-I Binding Grooves

As discussed above, indications of peptide motions within the MHC-I binding groove can come from crystallographic structures. This can include the presence of multiple peptide conformations, missing electron density, or significant conformational changes occurring upon TCR binding. However, moving from structural indications of motion to more detailed, actual assessments of motion requires additional experiments.

The simplest approach is to use crystal structures as the basis for molecular dynamics (MD) simulations. MD simulations take the initial set of atomic coordinates and compute the time-dependent variations in structure using classical laws of motion and a “force field” that describes the interactions between atoms. With ever-growing improvements in computer hardware, MD simulations have advanced considerably, such that we are now seeing simulations of entire protein folding reactions on timescales extending beyond microseconds (32). MD simulations have been used extensively to study the motions of peptides bound to MHC-I proteins (15, 33–51), as reviewed recently by Freund and colleagues (52). MD simulations have the advantage of tracking actual motion in atomic detail, capturing different conformations as a function of time. In most cases, however, MD simulations are limited to capturing motions that occur on fast timescales, and of course are virtual, with confidence in the results depending on the quality of the initial structure and a wide range of parameters that can be tuned to optimize speed versus accuracy.

Direct experimental measurements of peptide motions are less common than MD simulations, owing to the challenges of producing and working with peptide/MHC-I complexes. NMR spectroscopy is the gold standard for experimentally monitoring protein motion in atomic detail. NMR can provide motional detail across fast (picosecond to nanosecond) and slow (millisecond or greater) timescales and in some cases, can give insight into structural aspects of motion (e.g., amplitudes and directions). However, NMR on proteins the size and complexity of peptide/MHC complexes is technically challenging and of low throughput. Correspondingly, NMR has seen less use in studying peptide/MHC-I complexes (53–58). In two recent NMR studies, peptides bound to MHC-I proteins were shown to interconvert between conformations seen in different crystallographic structures (53, 56), confirming the expectation that crystallographically observed conformational changes reflect the propensity to move.

Fluorescence spectroscopy is commonly used to study protein motion, and it has seen some use in studying peptide/MHC-I complexes (35, 45, 59–62). In one notable study, fluorescence polarization was used along with MD simulations to show that allelic variations in HLA-B27 led to differences in peptide motions (35). A limitation of fluorescence is a reliance on bulky fluorescent labels, which can perturb structure and dynamics (63) [though in at least one case, intrinsic tryptophan fluorescence has been used to study MHC-I behavior (62)]. Infrared spectroscopy is another approach that may prove to be of use in studying the motions of peptides bound to MHC proteins, as it can report on fast motions using approaches analogous to NMR, but with reporters that are less perturbative—and sometimes non-perturbative in the case of C-D probes—to the native structure and dynamics of interest (64, 65).

## Peptide Motions and Antigenicity: Modified Peptides and Tumor Neoepitopes

From the discussion thus far, it is evident that motion of peptides in MHC-I binding grooves will impact TCR binding, influencing association rates, entropic penalties, or both. Although exceptions are known, in general peptide antigenicity scales with the affinity of the TCR for the peptide/MHC complex, with thresholds at the high and low limits (30, 66, 67). All other factors being equal, increasingly mobile peptides will be recognized more weakly by TCRs, with correspondingly reduced antigenicity.

The clearest example of the influence of peptide motion on antigenicity comes from the family of MART-1 tumor antigens and associated variants. The MART-1_27–35_ nonamer (sequence AAGIGILTV) is weakly immunogenic, attributed to its weak binding to HLA-A2 (68, 69). Weak binding stems from the presence of a suboptimal alanine at the first primary anchor position (peptide position 2). Modification of peptide primary anchors is a well-known strategy for improving peptide binding, and in some cases, antigenicity (70). Curiously, replacing the second alanine of the MART-1_27–35_ nonamer with leucine eliminates antigenicity with multiple T cell clones, despite strengthening MHC binding. The crystal structure of the anchor modified variant bound to HLA-A2 showed multiple conformations of the peptide in the binding groove, compared to a single conformation with the native, unmodified peptide (15). NMR confirmed the crystallographic data, showing the modified peptide indeed sampled multiple conformations (38). Exploration of peptide motions using MD simulations suggested the multiple conformations were attributable to rapid, nanosecond motions in the backbone of the modified peptide that were absent from the native peptide (comfortingly, the MD simulations recapitulated the conformations that were seen crystallographically). Binding experiments with recombinant TCRs confirmed the more dynamic modified peptide was in fact recognized more weakly, explaining its reduced antigenicity.

The results with the MART-1 nonamer suggest that screening for changes in peptide motion may be useful in helping predict antigenicity. One area where this may be particularly helpful is in predicting the antigenicity of “neoepitopes” arising from mutations present in tumor genomes. Neoepitopes are of considerable interest in cancer immunotherapy (71). However, predictive algorithms historically used in identifying immunodominant epitopes from viral genomes have performed poorly in predicting neoepitope antigenicity (37, 72). Toward this end, we used structural modeling and MD simulations to investigate a small set of neoepitopes and their wild-type counterparts bound to the mouse MHC-I protein H-2K^d^. Although only a small number of peptides were examined, there was a positive relationship between mutations which led to reduced peptide motions and antigenicity (37). The relationship between peptide rigidity and immunogenicity is consistent with the results seen with the MART-1 nonamer, as well as the theoretical considerations noted above: mutations that reduce motion should enhance T cell recognition by increasing TCR association rates and decreasing entropic costs for binding. Other factors are undoubtedly important to consider in neoantigen immunogenicity (peptide processing, MHC binding, amino acid composition, etc.), but incorporating structural modeling and predictions of how mutations alter fast peptide dynamics may provide another layer of sophistication for prediction methods and increase the likelihood of identifying those neoepitopes most likely to induce tumor rejection.

## Extension of Peptide Dynamics into MHC-I Proteins

Despite the common illustrations that render peptides and MHC-I proteins as distinct components (e.g., Figure 1), peptides are usually closely packed within MHC-I binding grooves [excluding those that are unusually long and bulge extensively (7, 8, 73–75)]. Therefore, it should not be surprising that peptide features can influence features of the peptide-binding groove. The possibilities for peptide-dependent structural shifts in MHC-I α helices were first noted in 1996 (76). More recently, we performed a comprehensive analysis of 51 structures of peptide/HLA-A2 complexes and found systematic deviations in the width of the peptide-binding groove and the bends and positions of both the α1 and α2 helices (26).

If there are peptide-dependent structural MHC-I shifts, there must be peptide-dependent MHC-I motions. Indeed, this was observed in 2009 when recognition of the Tax and Tel1p peptides by the A6 TCR was shown to yield different conformations in the α2 helix of HLA-A2 (34). It was shown that the α2 helix moved differently on the nanosecond timescale with the two peptides bound, which contributed to the structural shift seen with TCR binding to Tel1p versus Tax. The motions underlying the helix conformational change fit were found to be dependent on peptide structural features, and were correlated with differential peptide dynamics. Importantly, the different positioning of the MHC α2 helix was necessary for optimal interactions with the TCR, demonstrating that the “tuning” of HLA-A2 motions by the Tax and Tel1p proteins was indeed functionally important. Peptide-dependent alterations in the properties of the MHC-I α2 helix have been seen in other cases (77), suggesting that this region of the protein may be particularly sensitive to features of different peptides.

Returning to the MART-1 peptide system, *via* MD simulations, alteration of the MART-1_27–35_ nonamer was shown to alter the fluctuations of the α1 and α2 helices (38). More recently, fluorescence anisotropy and hydrogen/deuterium exchange was used to show that peptide-dependent tuning of MHC-I protein dynamics is a general phenomenon, not limited to particular peptides or a single site in the peptide-binding domain (26). Further evidence has been seen in a NMR study of HLA-B35, where it was shown that different peptides altered MHC sampling of minor conformations that had not been observed crystallographically (58). A recent analysis of temperature factors in peptide/MHC-I structures provided additional data and highlighted regions which might be particularly susceptible to include the α1 and α2 helices, as well as regions in the α3 domain and even β_2_-microglobulin (52). Thus, there is a structural and dynamic “extension of antigenicity” from the peptide to the MHC protein, with significant potential to influence TCR and T cell recognition. There is also evidence that allele-specific variations in different MHC-I proteins can modulate the susceptibility for influencing protein motions (35, 36, 44, 47–49, 78), suggesting a previously unrecognized complexity in how MHC genetics can influence antigenicity.

How can different peptides modulate MHC protein dynamics? Although they may be structurally similar, at the atomic level different peptides will form many different interactions with an MHC protein, as shown in Figure 2B. The different interatomic interactions will be of disparate energies; even a hydrogen bond between two identical atoms will be of different strengths if the geometries differ (26, 76). Thus, the free energies of structural substates will vary with different peptides bound. This is diagrammed in Figure 2C, which shows a peptide/MHC “folding funnel” leading from unfolded/unassembled protein to the assembled peptide/MHC-I native state. Different peptides will tune the energy landscape of the assembled complex as discussed above. In other words, the levels of the wells describing different structural states will move up or down depending on the identity of the peptide. This will change the heights of the barriers between them, altering the rates of conformational exchange, i.e., changing protein motions. Moreover, with different energies for different substates, the overall entropy of the protein will change, potentially leading to different entropy changes upon TCR binding and impacting binding affinity. Note that as crystallographic structures do not show energy and do not provide clear insights into structural substates, alterations in peptide/MHC dynamics can occur even in the absence of any apparent crystallographic differences (58). This same phenomenon explains how allelic variations can change peptide–protein dynamics: amino acid differences between MHC variants result in altered protein–peptide contacts, affecting the free energies of structural substates and with corresponding impacts on protein dynamics.

Peptide-dependent protein motion could also explain observations of peptide-dependent binding of other proteins to MHC-I, such as NK receptors (79). Indeed, it is believed that alterations in protein fluctuations is a key mechanistic component of peptide loading and exchange, perhaps in conjunction with partial peptide dissociation and the corresponding alterations in MHC dynamics (42, 44–46, 48). In classic biochemical terms, peptides are acting as allosteric effectors, modifying the binding of these other proteins and subsequent immunological functions. The underlying mechanisms by which peptides allosterically alter motions at remote sites is not clear, but could involve either discrete pathways of motion or more global alterations of the protein energy landscape and subsequent dynamics (80).

## Dynamics in Class II MHC (MHC-II) Proteins

The discussion above has centered on peptide motion within MHC-I proteins. Are there parallels with class II MHC proteins? The theoretical possibility of course exists, and the biochemical and functional implications are the same: peptide motion in MHC-II binding grooves can influence the binding of TCRs and other receptors, and peptides and MHC-II allelic variations have potential to influence MHC-II protein motions in functionally significant ways. MHC-I and MHC-II proteins are structurally homologous. However, in MHC-II, the peptide termini extend from the binding groove and, therefore, MHC-II-bound peptides are more extended, lack the bulges seen with peptides bound to MHC-I, and are more hydrogen-bonded to the protein (1). Likely for these reasons, large-scale peptide conformational changes upon TCR binding are seen less frequently with MHC-II systems (although there is less structural data for MHC-II). However, TCRs also bind peptide/MHC-II complexes with slow kinetics and occasionally unfavorable entropy changes (30, 31). Hairpin-style secondary structures have been seen in class II-presented peptides (81) and flanking regions of class II-presented peptides that lie outside of the groove can impact TCR binding, possibly due to dynamic effects (82). Therefore, although the data are less apparent, motions of peptides bound to MHC-II systems should also be considered when taking stock of the physical influences on TCR binding and peptide antigenicity. Furthermore, there is clear evidence for peptide influences on the motion of the MHC-II molecule, particularly in regions that interact with the peptide-exchange catalyst HLA-DM, as well as a fundamental role for protein motion in MHC-II peptide-exchange itself (83–86). Thus, in addition to having converged on fundamentally similar structures and functions, MHC-I and MHC-II protein functions may be similarly influenced by—and take advantage of—peptide and protein motional properties.

## Conclusion

Structural biology of peptides presented by MHC proteins has transformed our understanding of immunology, with impacts ranging from our understanding of fundamental immune mechanisms to the design and optimization of vaccines. However, considering peptide/MHC complexes not as static structures but as molecules that move and breathe has opened new avenues of investigation and shed new light on factors that impact immunogenicity. The mobility of peptides within MHC protein binding grooves can impact antigen immunogenicity, regardless of whether mobility or conformational variability is apparent from crystallographic structures. This is relevant in vaccine design, as well as predicting immunogenic epitopes from pathogen and tumor genomes. Efforts to predict peptide mobility may be particularly helpful in screening cancer neoepitopes, which have proven particularly challenging for predictive immunology. Peptides also “tune” the mobility of MHC proteins themselves, contributing to antigenicity and affecting interactions with other proteins. The latter can include the machinery of peptide loading and exchange as well as other activating and inhibitory immune receptors. Broader characterization of the energy landscapes of peptide/MHC complexes and the resulting functional consequences is clearly one of the next frontiers in structural immunology.

## Author Contributions

The manuscript was developed and written by all three authors.

## Conflict of Interest Statement

The authors declare that the research was conducted in the absence of any commercial or financial relationships that could be construed as a potential conflict of interest.
